# Pharmacodynamics of piperacillin/tazobactam against *Pseudomonas aeruginosa*: antibacterial effect and risk of emergence of resistance

**DOI:** 10.1093/jacamr/dlae108

**Published:** 2024-07-12

**Authors:** Amy A Carson, Karen E Bowker, Marie Attwood, Alan R Noel, Alasdair P MacGowan

**Affiliations:** Infection Sciences, Pathology Quarter, Southmead Hospital, Westbury-on-Trym, Bristol BS10 5NB, UK; Bristol Centre for Antimicrobial Research & Evaluation (BCARE), Severn Infection Sciences, Pathology Quarter, Southmead Hospital, Westbury-on-Trym, Bristol BS10 5NB, UK; Bristol Centre for Antimicrobial Research & Evaluation (BCARE), Severn Infection Sciences, Pathology Quarter, Southmead Hospital, Westbury-on-Trym, Bristol BS10 5NB, UK; Bristol Centre for Antimicrobial Research & Evaluation (BCARE), Severn Infection Sciences, Pathology Quarter, Southmead Hospital, Westbury-on-Trym, Bristol BS10 5NB, UK; Bristol Centre for Antimicrobial Research & Evaluation (BCARE), Severn Infection Sciences, Pathology Quarter, Southmead Hospital, Westbury-on-Trym, Bristol BS10 5NB, UK

Piperacillin/tazobactam has a broad spectrum of antibacterial activity, which makes it suitable therapy for severe sepsis in the hospital setting, especially in critical care.^1^ Despite this, there remains a paucity of published preclinical pharmacodynamic (PD) characteristics of piperacillin/tazobactam to aid our optimization of dosing.^2^ Studies indicate that the pharmacokinetic/PD (PK/PD) index of effectiveness for β-lactams is the percentage of time that the free drug concentration remains greater than the MIC, *fT*_>MIC_.^3^ A PD index target of *fT*_>MIC_ ≥ 50% is widely used to define antibacterial breakpoints for piperacillin/tazobactam, but the appropriate PD index targets for critical care patients is less clear.^4^ In critical care, alternative β-lactam targets have been employed, such as exceeding the β-lactam trough concentration by 4 ×  the pathogen MIC.^5^ PD factors have also led to the adoption of prolonged or continuous infusion therapies of piperacillin/tazobactam to treat aerobic Gram-negative rods and, most especially, *Pseudomonas aeruginosa* infection.^6–8^ However, there are burgeoning concerns regarding these ‘*supratherapeutic*’ doses, with the prevalence of β-lactam toxicity in the critical care environment thought to be underestimated.^9,10^ Here we aim to increase our preclinical data and evaluate the activity of piperacillin/tazobactam against *P. aeruginosa*, focusing on the *fT*_>MIC_ target for antibacterial effect (ABE) and emergence of resistance (EOR).

Four clinical strains of *P. aeruginosa* were used: 45966 (piperacillin/tazobactam MIC 4 mg/L); 46042 (MIC 6 mg/L); 27853 (MIC 4 mg/L); and 46172 (MIC 6 mg/L). MICs were obtained following EUCAST guidelines.^11^ Clinical strains were obtained from North Bristol NHS Trust and tested alongside the QC ATCC strain 27853 (MIC 4 mg/L). *fT*_>MIC_ dose ranging from 0% to 100% *fT*_>MIC_ for piperacillin/tazobactam was tested against these strains in a dilutional single compartment *in vitro* PK model (IVPKM). A pharmacy preparation of piperacillin/tazobactam (4 g/0.5 g; Milpharm, UK) was used for both dose ranging and piperacillin/tazobactam dose escalation simulations, with *t*_½_ = 1 h, 8 hourly, and a minimum of eight experiments per strain performed to simulate 0%–100% *fT*_>MIC_. The *fT*_>MIC_ ratios were based on the piperacillin concentrations with a ratio of 1:0.125 piperacillin:tazobactam. Mueller–Hinton broth (100%; Thermo Fisher, Basingstoke, UK) was used in all experiments. Nutrient agar plates were used to recover cfu enumerations per timepoint, 0–8 h, and every subsequent 24 h increment per IVPKM/per simulation, and blood agar plates were poured containing multiples of piperacillin/tazobactam MIC, measured in mg/L every 24 h increment from T0 to determine EOR. The inoculum was 10^6^ cfu/mL and experiments were performed over 72 h.

The relationship between *fT*_>MIC_ and ABE were described by log reduction in viable count at 24, 48 and 72 h. This was done using a Boltzmann sigmoid *E*_max_ equation with the software package GraphPad Prism: y = bottom + (top−bottom)/{1 + exp(V_50_−x)/slope]} (San Diego, CA, USA). EOR was assessed by changes in population profile from baseline at time 0 and 24 h by culture onto media containing ×4 and ×8 piperacillin/tazobactam MIC. Viable counts (log cfu/mL) were determined over the 72 h of piperacillin/tazobactam exposure.

At 24 h, the piperacillin/tazobactam *fT*_>MIC_ for ABE static effect was 39.8% ± 7.6%, for a −1 log drop was 51.7% ± 11.7%, and for a −2 log drop was 61.6% ± 17.7%; −3 log drop was not achieved in two of the four strains. At 48 h, *fT*_>MIC_ for static effect was 76.5% ± 14.2%, for a −1 log drop was 81.7% ± 10.2%, −2 log drop was not achieved (>100%), and for a −3 log drop was >100%. At 72 h, *fT*_>MIC_ for static effect was 86.6% ± 3.1%, for a −1 log drop was 89.5% ± 4.4%, −2 log drop was not achieved (>100%), and for −3 log drop was >100% (Table 1).

**Table 1.** %*fT*_>MIC_ piperacillin/tazobactam targets for static and bactericidal antipseudomonal effects after 24, 48 and 72 h exposure

	%*fT*_>MIC_ target				
Strain	45966 (MIC 4 mg/L)	46042 (MIC 6 mg/L)	27853 (MIC 4 mg/L)	46172 (MIC 6 mg/L)	Mean ± SD
24 h					
Static	32.5	52.0	40.3	34.2	39.8 ± 8.8
−1 log drop	36.9	69.1	53.7	47.0	51.7 ± 11.7
−2 log drop	40.5	83.9	67.8	60.4	61.6 ± 17.7
−3 log drop	>100	>100	91.3	95.3	Not achieved
48 h					
Static	58.4	97.6	71.5	78.5	76.5 ± 14.2
−1 log drop	73.8	98.7	73.8	80.5	81.7 ± 10.2
−2 log drop	89.9	99.5	>100	83.2	Not achieved
−3 log drop	>100	>100	>100	>100	>100
72 h					
Static	85.9	NF	89.9	83.9	86.6 ± 3.1
−1 log drop	93.3	NF	90.6	84.6	89.5 ± 4.4
−2 log drop	>100	NF	92.6	85.2	Not achieved
−3 log drop	>100	NF	>100	>100	>100

The risk of EOR, as indicated by the recovery of resistant isolates on ×4 MIC or ×8 MIC plates exhibited an inverted U pattern, demonstrating that lower doses exhibit less selective pressure. Initially the lower dose produced a small amount of resistance, and as this dose increased so did the EOR; this then continued up to a critical point whereby the bacteria were overcome by the higher concentrations of piperacillin/tazobactam. At 24 h, EOR steadily rises with the maximum risk of EOR being at an *fT*_>MIC_ of 40%–60%, then decreases at higher targets, with *fT*_>MIC_ of >60%–80%. The complete population profiles for ×4 MIC and ×8 MIC recovery plates are shown in Table S1 (available as Supplementary data at *JAC-AMR* Online).

In conclusion, an *fT*_>MIC_ of 50% for piperacillin/tazobactam is associated with a −1 log reduction in bacterial counts of *P. aeruginosa* after 24 h exposure. *fT*_>MIC_ targets of >80% are required for static or −1 log kill over 72 h. *fT*_>MIC_ in the range of 40%–60% maximally amplifies resistance at 24 h, decreasing at higher targets of *fT*_>MIC_ > 60%.

Our *in vitro* data for piperacillin/tazobactam align well with the current tenet of a PD index target of *fT*_>MIC_ ≥ 50% for reduction in bacterial counts. Less studied is EOR; here, selection of resistance is most pronounced in the range of 40%–60% *fT*_>MIC_ , and so prevention of EOR *in vitro* requires targets of *fT*_>MIC_ > 60%. This data suggest that while the conventional dose of piperacillin/tazobactam can be used successfully to treat *P. aeruginosa* infection, dose adjustment to achieve trough concentrations of >4 ×  pathogen MIC may be unnecessary. In addition, it may be associated with unintended toxicity.^5^ To prevent EOR *in vitro* requires targets of *fT*_>MIC_ > 60%.

This was an *in vitro* experiment, and the authors acknowledge the challenges in translating this to an *in vivo*/clinical recommendation.

## Supplementary Material

dlae108_Supplementary_Data

## Supplementary data

Table S1 is available as Supplementary data at *JAC-AMR* Online.

## References

[1] Barton GJ, Morecroft CW, Henney NC. A survey of antibiotic administration practices involving patients with sepsis in UK critical care units. Int J Clin Pharm 2020; 42: 65–71. 10.1007/s11096-019-00938-9 31728749 PMC7162826

[2] Hoo GSR, Liew YX, Kwa AL-H. Optimisation of antimicrobial dosing based on pharmacokinetic and pharmacodynamic principles. Indian J Med Microbiol 2017; 35: 340–6. 10.4103/ijmm.IJMM_17_278 29063877

[3] Lodise TP, Lomaestro BM, Drusano GL. Application of antimicrobial pharmacodynamic concepts into clinical practice: focus on β-lactam antibiotics: insights from the Society of Infectious Diseases Pharmacists. Pharmacotherapy 2006; 26: 1320–32. 10.1592/phco.26.9.1320 16945055

[4] Roberts JA, Paul SK, Akova M et al DALI: defining antibiotic levels in intensive care unit patients: are current β-lactam antibiotic doses sufficient for critically ill patients? Clin Infect Dis 2014; 58: 1072–83. 10.1093/cid/ciu027 24429437

[5] Guilhaumou R, Benaboud S, Bennis Y et al Optimization of the treatment with beta-lactam antibiotics in critically ill patients—guidelines from the French Society of Pharmacology and Therapeutics (Société Française de Pharmacologie et Thérapeutique—SFPT) and the French Society of Anaesthesia and Intensive Care Medicine (Société Française d’Anesthésie et Réanimation—SFAR). Crit Care 2019; 23: 104. 10.1186/s13054-019-2378-9 30925922 PMC6441232

[6] Babich T, Naucler P, Valik JK et al Ceftazidime, carbapenems, or piperacillin-tazobactam as single definitive therapy for *Pseudomonas aeruginosa* bloodstream infection: a multisite retrospective study. Clin Infect Dis 2020; 70: 2270–80. 10.1093/cid/ciz668 31323088

[7] Fawaz S, Barton S, Nabhani-Gebara S. Comparing clinical outcomes of piperacillin-tazobactam administration and dosage strategies in critically ill adult patients: a systematic review and meta-analysis. BMC Infect Dis 2020; 20: 430. 10.1186/s12879-020-05149-6 32563242 PMC7305614

[8] Thabet P, Joshi A, Macdonald E et al Clinical and pharmacokinetic/dynamic outcomes of prolonged infusions of beta-lactam antimicrobials: an overview of systematic reviews. PLoS One 2021; 16: e0244966. 10.1371/journal.pone.0244966 33481817 PMC7822342

[9] Roger C, Louart B. Beta-lactams toxicity in the intensive care unit: an underestimated collateral damage? Microorganisms 2021; 7: 1505. 10.3390/microorganisms9071505 PMC8306322 34361942

[10] Barreto EF, Webb AJ, Pais GM et al Setting the beta-lactam therapeutic range for critically ill patients: is there a floor or even a ceiling? Crit Care Explor 2021; 6: e0446. 10.1097/CCE.0000000000000446 PMC8202642 34136822

[11] EUCAST . Standard operating procedure 10.2: MIC distributions and the setting of epidemiological cutoff (ECOFF) values. https://www.eucast.org/fileadmin/src/media/PDFs/EUCAST_files/EUCAST_SOPs/2021/EUCAST_SOP_10.2_MIC_distributions_and_epidemiological_cut-off_value__ECOFF__setting_20211202.pdf.

