# Supplementary material for: Pharmacodynamics of piperacillin/tazobactam against Pseudomonas aeruginosa: antibacterial effect and risk of emergence of resistance
Source: JAC Antimicrob Resist. 2024 Jul 12;6(4):dlae108. doi: 10.1093/jacamr/dlae108 (PMC11242448; doi:10.1093/jacamr/dlae108)
Supplement: dlae108_Supplementary_Data [file dlae108_supplementary_data.docx]

**Supplementary data**

**Figures S1&S2: Changes in *P.aeruginosa* population profiles related to piperacillin/tazobactam exposure**

**Table S1: Population profiles for *P.aeruginosa* after exposure to piperacillin-tazobactam at a range of %*fT*>_MIC_ on x4MIC or x8MIC recovery plates at 24h, 48h and 72h**

|  | **24h** | | | | **48h** | | | | | **72h** | | | | | |
| --- | --- | --- | --- | --- | --- | --- | --- | --- | --- | --- | --- | --- | --- | --- | --- |
| **%*fT*>_MIC_ (%)** | MIC x 4 | | MIC x8 | | MIC x 4 | | MIC x 8 | | MIC x 4 | | | MIC x 8 | | |  |
|  | No. of experiments with growth on recovery medium and % | Mean bacterial count on recovery medium (log_10_ cfu/mL) ± SD | No. of experiments with growth on recovery medium and % | Mean bacterial count on recovery medium (log_10_ cfu/mL) ± SD | No. of experiments with growth on recovery medium and % | Mean bacterial count on recovery medium (log_10_ cfu/mL) ± SD | No. of experiments with growth on recovery medium and % | Mean bacterial count on recovery medium (log_10_ cfu/mL) ± SD | No. of experiments with growth on recovery medium and % | | Mean bacterial count on recovery medium (log_10_ cfu/mL) ± SD | No. of experiments with growth on recovery medium and % | Mean bacterial count on recovery medium (log_10_ cfu/mL) ± SD |  |  |
| **0** | 0/6 (0%) | 1.9 | 0/6 (0%) | 1.9 | 0/6 (0%) | 1.9 | 0/6 (0%) | 1.9 | 2/6 (33%) | | 2.8 | 0/6 (0%) | 1.9 |  |  |
| **>0 - 20** | 4/7 (57%) | 2.9 ± 0.8 | 2/7 (29%) | 2.4 | 4/7 (57%) | 3.1 ± 0.9 | 2/7 (29%) | 2.6 | 6/7 (100%) | | 4.2 ± 1.6 | 5/7 (100%) | 4.0 ± 1.1 |  |  |
| **>20 - 40** | 6/8 (75%) | 4.3 ± 0.7 | 5/8 (63%) | 3..1 ± 1.1 | 8/8 (100%) | 5.2 ± 0.8 | 8/8 (100%) | 4.3 ± 0.5 | 8/8 (100%) | | 6.4 ± 1.0 | 8/8 (100%) | 4.8 ± 0.8 |  |  |
| **>40 - 60** | 12/14 (86%) | 4.5 ± 1.1 | 10/14 (71%) | 3.75 ± 0.9 | 13/13 (100%) | 5.7 ± 1.1 | 13/13 (100%) | 4.6 ± 0.6 | 13/13 (100%) | | 6.2 ± 1.1 | 13/13 (100%) | 4.9 ± 1.2 |  |  |
| **>60 - 80** | 4/6 (67%) | 3.4 ± 1.0 | 2/6 (33%) | 2.7 | 6/6 (100%) | 4.9 ± 1.0 | 5/5 (100%) | 4.9 ± 1.0 | 5/5 (100%) | | 6.5 ± 1.0 | 6/6 (100%) | 5.6 ± 1.6 |  |  |
| **>80 - 100** | 2/10 (20%) | 3.1 | 1/10 (10%) | 2.7 | 4/4 (100%) | 4.0 ± 1.1 | 3/3 (100%) | 3.9 ± 1.0 | 5/5 (100%) | | 6.2 ± 1.1 | 5/5 (100%) | 5.4 ± 1.5 |  |  |
